# Comparative Analysis of Quantitative Methods for *Campylobacter* spp. Quantification: ISO 10272-2:2017, Tempo^®^ and Real-Time PCR in Refrigerated and Frozen Turkey Cuts

**DOI:** 10.3390/foods13213359

**Published:** 2024-10-23

**Authors:** Carlos Alberto Führ, Audecir Giombelli, Marisete Fochesatto Cerutti, Guiomar Pedro Bergmann, Liris Kindlein

**Affiliations:** 1 Postgraduate Program in Foods of Animal Origin, Federal University of Rio Grande do Sul, Porto Alegre 91540-000, Brazil; carlos.fuhr@seara.com.br; 2 Seara Alimentos Ltda, Itajaí 88305-030, Brazilmarisete.cerutti@seara.com.br (M.F.C.); 3 Department of Veterinary Medicine, Federal University of Rio Grande do Sul, Porto Alegre 91540-000, Brazil

**Keywords:** *Campylobacter*, ISO 10272-2, real-time PCR, TEMPO^®^ CAM, turkey meat

## Abstract

New technologies for more effective microbiological assays are being adopted by the food industry to intervene more rapidly in its production chain. The aim of this study was to evaluate the alternative methods of TEMPO^®^ CAM and real-time PCR (rtPCR) Biotecon^®^ in comparison with the ISO 10272-2:2017 reference method for *Campylobacter* spp. quantification in turkey meat, aiming to validate a quick and easily replicable method in these meat matrices. A total of 416 samples were analyzed over a one-year period. The TEMPO^®^ methodology showed inadequate performance with a significant difference (*p* < 0.05) compared with the reference methodology; therefore, its use was not recommended for turkey meat matrices. However, the performance of the rtPCR Biotecon^®^ methodology showed adequate performance with no significant difference (*p* > 0.05), and its use was recommended in turkey meat matrices. The study was limited to exclusive research in turkey meat matrices, and expansion of the research into other matrices is recommended to verify whether the behavior of alternative methodologies is similar. The findings of this study illustrate the necessity for a thorough and comprehensive evaluation during the implementation of alternative methodologies that may potentially supplant conventional approaches.

## 1. Introduction

Animal-protein-based products are deemed to be the main cause of foodborne bacterial diseases [1].

Reports of occurrences of campylobacteriosis in humans caused by poultry-based foodstuffs contaminated by *Campylobacter* spp. have increased considerably in many countries [2]. Raw poultry meats may be a source of contamination by *Campylobacter* spp. because the micro-organism may live in the intestine of healthy birds [3], and *Campylobacter* spp. is the major cause of bacterial gastroenteritis worldwide [4]. Therefore, precautions to mitigate the agent should be taken from the field up to the manufacturing and processing lines of the final product.

According to Tack et al. [5], during 2019, the overall incidence per 100,000 population was highest for *Campylobacter* spp. (19.5) and compared with 2016–2018, the incidence in 2019 increased significantly for *Campylobacter* spp. (13%). However, in Europe in 2020, the most commonly reported zoonosis in humans was campylobacteriosis, and the cost to the public health system, as well as in terms of loss of productivity, in the European Union is estimated at roughly EUR 2.4 billion annually [3].

In developing countries, the information on foodborne diseases is scarce owing to the fact that the health surveillance systems provide incomplete data or that they even provide no data at all [6]. Furthermore, the outbreaks are often underreported, and information is therefore lacking because it is not part of the culture for the population to inform the competent authorities; health authorities possess poorly grounded data because they lack the means to detect diarrheal diseases [6], and the logistics around sample-shipping and laboratory methodologies are limited.

Many domestic poultry species, such as hens, turkeys, ducks and geese, as well as wild birds, are often infected by thermophilic *Campylobacter* spp., particularly *Campylobacter jejuni* and *Campylobacter coli* [7]. The pathogens have been extensively studied in hens, but little information is available on turkeys [8]; however, turkey meat possesses the potential for transmission, and according to the literature, it has already been implicated in foodborne outbreaks [9]. Despite the increased production and consumption of turkey meat in recent years and the high prevalence of *Campylobacter* spp. detected in turkey meat sold in Europe (65.3%) [10], most studies on the epidemiology of *Campylobacter* spp. have focused mainly on the production of broiler chickens [11], and the sources of contamination for turkey carcasses are similar to those for chicken carcasses; however, reports on the involvement of turkey meat in foodborne diseases (FBDs) and risks to public health are scarce [9].

Turkey meat cuts are usually presented chilled or frozen: chilling and freezing are processes that control microbial growth. The chilling and freezing processes are widely used methods for maintaining the sanitary quality of highly perishable animal foodstuffs; in the case of cuts of meat, they inhibit the growth of foodborne-disease-causing micro-organisms [12,13].

According to Gouvêa et al., the total mesophilic micro-organism count is one of the most important indicators for assessing the freshness of the meat [14]. For contamination caused by the slaughter process, particularly fecal contamination, as caused by intestinal rupturing, or external contamination of animals on the slaughter line, *Enterobacteriaceae* and *E. coli* are deemed the most suitable indicator organisms [15]. In Brazil, the hygiene criteria for intensive poultry raising are represented by evaluation for the presence of *Salmonella* on chicken carcasses after the pre-chill step in the national pathogen reduction program [16]. Bacteria normally require ideal time and temperature conditions—that is to say, an energy source (oxygen) and a suitable relative humidity and temperature—in order to survive and reproduce [14]. Stella et al. concluded that chilling may be deemed a useful intervention to address *Campylobacter* spp. in an overall food safety management system [17]. Maziero and Oliveira, on the other hand, reported that chilling or freezing procedures do not fully guarantee food safety in the case of *Campylobacter* spp. [18].

The poultry industry has grown quickly, and producing safe foodstuffs for consumers is a concern; therefore, constant enhancement of sanitary control measures on farms and in processing units is necessary in order to prevent these products from leading to the contamination of foods and the occurrence of foodborne diseases [19]. According to Seliwiorstow et al., the contamination of poultry meat by *Campylobacter* spp. in the slaughter process is influenced by the initial contamination displayed by the birds on arrival (both external contamination and the level of colonization of the cecum) and by the duration of the pre-slaughter feed withdrawal period [20]. During this period, there is a drastic reduction in the beneficial competitive microbiota, enabling the survival and proliferation of bacteria such as *Campylobacter* spp. because the intestinal contents become more moist and fluid, making them more liable to cause contamination [21]. Technology failures in the slaughter process, such as excessively low scalding water temperature, poorly adjusted plucking devices and inefficiencies in the evisceration step (gastrointestinal rupture), contribute to cross-contamination in the process. Franchin et al. describe the reduction in *Campylobacter* spp. counts achieved with good sanitary and hygiene controls practiced throughout the farm-to-packaging process, efficient Good Manufacturing Practices (GMP) programs and Hazard Analysis and Critical Control Point (HACCP) programs in slaughterhouses [22].

According to Stella et al., *Campylobacter* spp. is playing an increasingly important role as a foodborne pathogen, and domestic poultry is the main vehicle for infection, leading to slaughterhouses introducing control measures in recent years [17]. It is therefore of the utmost importance that barriers are applied strategically, including refrigeration in the cold chain, enabling food business operators to meet process hygiene criteria, thus preventing the delivery of highly contaminated meats [17].

Self-control programs, good manufacturing practice programs, sanitation standard operating procedures and hazard analysis and critical control point programs have all been introduced in order to ensure the safety and quality of food products [23]. The main goal of such programs is to promote hygiene in the following areas: the environment, utensils, employees and equipment, checking critical control points and keeping the process under control, so that safe foods may be offered [24].

Quality assurance monitoring and the guarantee of product integrity are fundamental factors for consumer safety, and in order to obtain good results that may contribute to the slaughterhouses’ quality indicators, one premise is the use of methodologies that are both reliable and efficient for quantifying *Campylobacter* spp. Rapid methods are more advantageous owing above all to the time taken to obtain the results, since microbiological tests take a long time to present a conclusive result; therefore, rapid methods enable costs to be controlled and contamination events to be identified in advance [25].

According to Pacholewicz et al., laboratory detection and quantification of *Campylobacter* spp. are an obstacle [26]. Quantification is a challenge, and one of the main reasons is that because of cold stress or oxygen stress during storage in retail, the agent loses its ability as a bacterial culture [26]. Although the conventional culturing methodology is still a benchmark for detecting and quantifying *Campylobacter* spp., the consistency and reliability of several matrices obtained in poultry production are problematic [27], and technological innovation for detecting agents deemed to pose a threat to public health should be investigated.

*Campylobacter* spp. species are incapable of growing at a maximum temperature above 55 °C or below a temperature of 30 °C [28]. Species that are pathogenic to humans grow best at 42 °C [29], grow well in water activity of 0.997 and do not grow in water activity of less than 0.987 [30]. Additionally, they do not grow in media with a sodium chloride concentration above 3.5% or at 25 °C, and they are microaerophilic, meaning they require small amounts of oxygen (3–6%) and a 2–10% concentration of carbon dioxide in order for them to develop [29].

Despite being time consuming, the plate-count method is the standard reference for counting *Campylobacter* spp. on chicken skin. However, owing to special requirements for growth, not all cells can be recovered via conventional culture techniques [31]. The isolation of *Campylobacter* spp. needs to be more accurate than that employed for other foodborne pathogens owing to its raised sensitivity to oxygen and to oxidizing radicals, to water activity, to temperature changes and to the presence of viable but non-cultivable forms [30].

The efficiency of culture-dependent quantification is affected by growth conditions and by the physiological state of the bacteria [32], such that a low concentration of target organisms may prevent reliable detection and quantification [31]; however, traditional microbiological culture methods have evolved over time to include the use of selective media, the optimization of growth conditions and antibiotic support in order to reduce the species of accompanying micro-organisms [33].

The conventional methodology for detecting *Campylobacter* spp. typically involves plating on selective agar after a period of enrichment. However, despite advances in the techniques used for isolating this micro-organism, the current methodologies continue to face challenges that limit their efficiency [34]. In Europe, Regulation (EU) 2017/1495 [35] recommends the use of the ISO 10272-2 [36] reference methodology for the testing of *Campylobacter* spp. This methodology is designed to quantify thermotolerant *Campylobacter* spp. that are relevant to human health.

In Brazil, the only regulatory document pertaining to the monitoring of *Campylobacter* spp. in chicken meat is Memorandum nº 06/2018 [37]. This document applies exclusively to plants authorized for the European market and is in compliance with Regulation (EU) 2017/1495 [35]. In light of the necessity to monitor *Campylobacter* spp. in poultry farming and the reality that turkey remains an unmonitored poultry in Brazil, the absence of a monitoring program and a paucity of comprehensive data to assess the level of risk associated with this poultry make it imperative to evaluate and quantify *Campylobacter* spp. This will facilitate a deeper understanding of the extent of contamination and contribute to the enhancement of the quality of turkey meat products on the market.

New high-yield bacteriological testing technologies have recently been introduced by food-producing manufacturers in order to intervene more rapidly in their production chains using methods that provide quicker results [38]. The TEMPO^®^ system is a methodology that is more and more widely used in the food business, which values its semi-automatic process with a built-in sample scanner for the purposes of traceability and its speed of attaining a bacteriological count and identification without additional identification tests [39].

The TEMPO^®^ system is based on the MPN technique and comprises a card, a vial containing the culture medium and a fluorescent indicator. Samples are added to vials of dehydrated medium and introduced onto the cards in an automated vacuum chamber, where the inoculated medium is automatically transferred to the card containing 48 wells with three different volumes. Subsequently, the cards are removed from the vacuum chamber and incubated in accordance with the specifications for each micro-organism [39,40]. During the incubation period, the micro-organism undergoes hydrolysis of the substrate present in the culture medium, resulting in the generation of a fluorescent signal that is detected by the TEMPO^®^ Reader. This device calculates the number of positive wells and expresses the results in CFU/g [39,40].

A study carried out by Yörük [41] considered TEMPO^®^ EC to be suitable for *E. coli* counting of spiked milk and milk product foods. A study by Torlak and Akan [42] showed that TEMPO^®^ STA is an efficient alternative method for the enumeration of coagulase-positive staphylococci in cheese.

Compared with traditional microbiology and enrichment techniques, molecular methods have the potential to be more sensitive and specific and obtain data more rapidly [27]. Real-time PCR (rtPCR) has been described as one of the most sensitive methods for detecting *Campylobacter* spp. [43]. A study carried out by Josefsen et al. [44] considered rtPCR to be a rapid tool for producing reliable quantitative data on viable *Campylobacter* spp. bacteria in chicken carcass.

rtPCR is a process whereby the target DNA is amplified and quantified concurrently within a single reaction. A specific primer set—one or two probes—and/or a fluorescent dye are employed to enhance the detection signals [45,46]. The amplified DNA is detected in real time as the reaction progresses rather than at the conclusion of the reaction. Real-time PCR (rtPCR) reduces the detection time in comparison with standard PCR and is capable of determining the absolute or relative number of bacteria in a variety of samples [46].

The present study aimed to assess two alternative methods against the reference ISO 10272-2:2017 [36] method using an assessment of the level of contamination in CFU/g of *Campylobacter* spp. in chilled and frozen turkey meat cuts. The TEMPO^®^ methodology (manufactured by bioMérieux^®^) with a TEMPO^®^ CAM kit and the real-time PCR (rtPCR) methodology (manufactured by Biotecon^®^) were used as alternative methods. Both of the alternative methodologies are specifically intended for quantifying *Campylobacter* spp.

## 2. Materials and Methods

### 2.1. Taking Samples and Defining the Sampling Number

The samples of chilled turkey cuts were all harvested aseptically in the slaughterhouse’s cutting room and packed in sterile packaging. The samples of frozen turkey cuts were all harvested in the freezing tunnel (−35 °C) and stored in freezers (at −20 °C) for at least 7 days. Every week, four chilled and four frozen samples were taken during cutting operations from both male and female turkeys over a 12-month period. In all, 208 chilled samples and 208 frozen samples were harvested from March 2022 to April 2023. The samples of chilled cuts were immediately stored in isothermal containers after they were taken and kept at a temperature between 1 °C and 8 °C prior to testing, which was carried out within 24 h of harvesting.

In order to calculate the sample size of the study and the desired confidence level, the formula described by Cannon [47] was used, which takes into account population size, the desired confidence level, the sensitivity and the expected prevalence of *Campylobacter* spp. The population size was calculated on the basis of the daily mean slaughter volume of 19,000 turkeys multiplied by the weekly number of slaughtering days. The desired confidence level and sensitivity were determined at 95%. The literature was examined (see Table 1) in order to define the expected percentage of prevalence, taking into account the prevalence of *Campylobacter* spp. in turkeys.

Samples were paired by collecting each cut from a different carcass, thus ensuring that the characteristics of the two samples were identical and that the representativeness of the chilled and frozen samples was as close as possible. It was thus suggested that the bacterial load variable should be more uniform, thereby enabling a more accurate comparison of the chilled and frozen conditions.

The variable of freezing was considered significant due to the findings of Rasschaert et al. [55], which indicated that freezing after the cooling stage resulted in a notable reduction in *Campylobacter* spp. counts. It is possible that additional variables beyond temperature may influence the colonization and transmission of *Campylobacter* spp. These may include flock size, age of the birds, initial count, quality of the water supply, presence of vectors (insects) and even environmental contamination [56]. However, these variables were not considered in the current research.

### 2.2. Sample Processing

All chilled samples were processed within 24 h after harvesting, and the frozen samples, having remained for at least seven days at −20 °C, were shipped to the laboratory, thawed at a controlled temperature (2–8 °C) and, having attained at least 2 °C, were analyzed within 24 h. The procedures of the ISO 6887-1:2017 [57] standard were followed in order to remove the sample portion from the samples. A 25 g aliquot was removed from each sample, and buffered peptone water (BPW) was used for dilution at 1% (BBV^®^, Valinhos, Brazil). The samples underwent homogenization in a homogenizer for approximately 30 s, the longest possible time that would prevent too much oxygen being incorporated into the sample.

### 2.3. Assay Quality Control

Assay quality control was performed on every date on which the samples were tested. For the negative control, the assay was carried out without the addition of the reference micro-organism; for the positive control, an aliquot of the working culture with the certified reference material (MRC Microbiologics^®^, Saint Cloud, MN, USA) of *Campylobacter jejuni* subsp. Jejuni, ATCC^®^ reference number 33560 TM, was used.

### 2.4. Campylobacter *spp.* Enumeration Using the Reference Method

A 1 mL aliquot of the solution (10^−1^ dilution) was removed from each sample and from the negative and positive controls that were fortified in advance with BPW 1% (BBV^®^, Valinhos, Brazil), and this was dispensed onto the surface of a 140 mm plate containing modified charcoal cefoperazone deoxycholate agar (mCCD agar; Merck^®^, Darmstad, Germany). For the 10^−2^ dilution, a 1 mL aliquot of the prepared solution was removed and passed to a test tube containing 9 mL BPW 0.1% (BBV^®^, Valinhos, Brazil), and then, a 1 mL aliquot was removed from the tube and dispensed onto the surface of a 140 mm plate containing mCCD agar (Merck^®^, Darmstad, Germany). Duplicate plating was performed for each dilution. After the aliquots were dispensed, the solution was spread on each plate using a sterile Drigalski loop (Cral^®^, Cotia, Brazil), and the plates were kept at room temperature for approximately 5 min, so that the culture medium could be completely absorbed. The plates were then promptly placed in a specific jar containing a microaerophilic environment (5% O_2_, 10% CO_2_ and 85% N_2_) using a microaerophilic generator (bioMérieux^®^, Marcy-l’Etoile, France) and were incubated in an incubator set to a temperature of 41.5 ± 1 °C for 40–48 h.

#### 2.4.1. Enumeration of the Plates After Incubation

After the incubation time for the samples, plates containing up to 150 colonies were selected. Plates containing presumptive metal-gray-colored colonies were numbered prior to the confirmation step. Up to 5 typical colonies (for plates containing more than 5 colonies) were subcultured onto Columbia blood agar (Laborclin^®^, Pinhais, Brazil) plates, and these were placed in a specific jar containing a microaerophilic environment (5% O_2_, 10% CO_2_ and 85% N_2_) using a microaerophilic generator (bioMérieux^®^, Marcy-l’Etoile, France) and were immediately incubated in an incubator set to a temperature of 41.5 ± 1 °C for 24–48 h.

#### 2.4.2. Confirmation of Typical Colonies: Oxidase Test

After incubation, based on the colonies isolated in blood agar, the oxidase test was performed first, removing one colony from the blood agar plate with a sterile loop, and the colony was streaked onto the surface of an oxidase strip (Laborclin^®^, Pinhais, Brazil), and the reading was taken after 10 s. If a color change occurs from a transparent mauve to violet or blue, the test indicates a positive reaction, since *Campylobacter* spp. are deemed to be oxidase-positive micro-organisms [58]. For the plates presenting a positive oxidase test, motility, morphology and aerobiotic growth tests were performed based on the colonies from the blood agar plate.

#### 2.4.3. Confirmation of Typical Colonies: Motility and Morphology Tests

Both the motility and morphology tests were performed, regardless of the confirmation technique. The motility test was performed by emulsifying a colony in two drops of semi-solid brucella broth (Merck^®^, Darmstad, Germany) on a glass slide. The colony was emulsified into the broth on the slide using an inoculation loop and was covered by a slip for microscopic (Nikon Instruments Inc., Tokyo, Japan) examination of motility with phase contrast. The motility characteristic was observed, where *Campylobacter* spp. presents as curved bacilli with corkscrew motility, as referenced in the literature [29]. 

Morphology was evaluated using Gram staining, whereby a drop of purified water was deposited on a glass slide, and one loop’s content of the culture from the blood agar plates was added to the water; following this, a smear of approximately 10–15 mm in diameter was produced, and afterward, the slide was left to dry naturally. Once dry, the smear was covered by a crystal violet solution (Laborclin^®^, Pinhais, Brazil) for 1 min, then washed immediately in purified water. Next, the slide was covered with Lugol solution (Laborclin^®^, Pinhais, Brazil) for 1 min and then rinsed in purified water. Alcohol (Merck^®^, Darmstad, Germany) was poured on the slide until the violet staining was no longer visible; then, it was rinsed gently with purified water to eliminate the alcohol. The slide was immediately covered with a solution of phenicated fuchsin (Merck^®^, Darmstad, Germany) for approximately 40 s; then, the slide was immediately washed gently in purified water and allowed to dry naturally. After this step, microscopic examination (Nikon Instruments Inc., Tokyo, Japan) could be performed using a 100× lens with immersion oil (Laborclin^®^, Pinhais, Brazil). In the interpretation of the test, it is worth noting that *Campylobacter* spp. present a morphological characteristic of small curved (“gull wing”) bacilli and a Gram-negative reaction [29]. The tinctorial staining of the bacteria is interpreted as Gram-negative when the coloring is between red and pink and as Gram-positive when the coloring is between blue and purple.

#### 2.4.4. Confirmation of Typical Colonies: Aerobiosis Test

The aerobiosis test was performed by isolating one colony using an inoculation loop onto a new Columbia blood agar slide (Laborclin^®^, Pinhais, Brazil), incubated in aerobiotic conditions at 25 ± 1 °C for 40–48 h. To confirm *Campylobacter* spp., the aerobiosis test must not present colony growth in the Columbia blood agar. If any colony growth occurs, then it is a different micro-organism because, according to Igwaran and Okoh, *Campylobacter* spp. needs microaerophilia [58].

In order to express the number of colonies as CFU/g, the final calculation was performed using the mean of the count of the two plates with the same dilution as that used for confirmation multiplied by the number of confirmed colonies and divided by the number of colonies that underwent confirmation (maximum of 5).

### 2.5. Campylobacter *spp.* Enumeration with the TEMPO^®^ Method Using the TEMPO^®^ CAM Kit 

After each sample was processed, the culture medium for the TEMPO^®^ CAM test (bioMérieux^®^, Marcy-l’Etoile, France) was reconstituted with 3.9 mL of sterile distilled water. Based on each sample enriched in BPW 1% (BBV^®^, Valinhos, Brazil) and on the positive and negative controls, one aliquot of 0.1 mL was removed from the sample into a CAM culture medium flask reconstituted in advance. After homogenization, the cards and culture medium tubes were correlated on the filling holder and transferred to the TEMPO^®^ Filler module (bioMérieux^®^, Marcy-l’Etoile, France) for automatic transfer of the CAM culture medium into the interior of the card.

Once the cards were filled and automatically sealed, they were transferred to an incubation stand, placed in a specific jar containing a microaerophilic environment (5% O_2_, 10% CO_2_ and 85% N_2_) using a microaerophilic generator (bioMérieux^®^, Marcy-l’Etoile, France) and were immediately incubated in an oven for 44–48 h set to a temperature of 41.5 ± 1 °C. After the incubation time had elapsed, the holders securing the cards were removed in order to attain room temperature and placed in the incubation holder in the compartment of the TEMPO^®^ Reader (bioMérieux^®^, Marcy-l’Etoile, France), which then read the cards, and the results were generated automatically, without any need for additional confirmation. The results of enumerations of *Campylobacter* spp. were saved automatically in the system’s software and were then obtained from the result screen.

### 2.6. Enumeration of Campylobacter *spp.* Using the Real-Time PCR Method (BIOTECON^®^)

Having been obtained from each sample that was pre-enriched in BPW 1% (BBV^®^, Valinhos, Brazil) and from the positive and negative controls, an aliquot of 1500 µL of the supernatant was transferred to a 2 mL tube, and 250 µL of reagent D (Biotecon^®^, Potsdam, Germany) was added, and this was then homogenized to remove the dead cells. The tubes were incubated for 10 min at room temperature in a D-Light (Biotecon^®^, Potsdam, Germany) apparatus under dark conditions, then for 5 min under light-exposure conditions. The tubes were centrifuged at 10,000 rpm to remove the supernatant; then, a solution of lysis buffer from the Starprep Two (Biotecon^®^, Potsdam, Germany) kit was subsequently added in order to apply the cell disruptor for 8 min. After cell disruption, the suspension was heated for 5 min in the 95–100 °C range, then immediately set aside to rest at room temperature for 1 min and then centrifuged at 13,000 rpm for 1 min to obtain the extracted DNA that was retained in the supernatant.

#### 2.6.1. Preparation of the Standard rtPCR Curve for Quantification

After each round, it was necessary to prepare a standard curve corresponding to the 10× dilution of the Foodproof (Biotecon^®^, Potsdam, Germany) quantification standard in six steps with Foodproof^®^ *Campylobacter* Dilution Buffer (Biotecon^®^, Potsdam, Germany). Each dilution step was prepared with a final volume of 100 µL, using 10 µL from the previous dilution step and 90 µL of Foodproof^®^ *Campylobacter* Dilution Buffer (Biotecon^®^, Potsdam, Germany); the first dilution was carried out with the standard without dilution, representing a concentration of 1,000,000 CFU/reaction, while the sixth (final) step had a dilution of 1:100,000, representing a concentration of 10 CFU/reaction. After the standards were prepared, the PCR tubes were prepared by adding 20 µL of the PCR mix that was prepared in advance, in accordance with the instructions accompanying the kit (Table 2), and 5 µL aliquots of the supernatant from the tubes containing the centrifuged samples, from the standards and from the positive and negative controls, were used. Immediately afterward, the tubes containing the samples were centrifuged and placed in a thermocycler Agilent AriaMX (Agilent^®^, Santa Clara, MN, USA) in order to amplify the DNA and adjusted to the following setup:

▪ Pre-incubation: 1 cycle;
▪ Step 1: 37 °C/4 min;
▪ Step 2: 95 °C/5 min;
▪ Amplification: 50 cycles;
▪ Step 1: 95 °C/5 s;
▪ Step 2: 60 °C/60 s—fluorescence detection.

#### 2.6.2. Evaluation of Results After DNA Amplification

After the run was completed, the software prepared a duplicate standard curve based on the standards and automatically compared this against the sample(s), returning a value that enabled the result to be calculated as colony-forming units (CFUs). A total of 13 reactions (tests) were used in order to assess whether the results were valid or otherwise. It was necessary to check the following aspects: the uniformity of the calibration points of the external standards; the occurrence and formation of a straight line; the presence of all these standard points on the curve; the absence of an amplification signal and curves above the threshold for the negative control; and amplification of all the negative samples in the HEX channel, which was the internal control (Table 3).

The DNA amplification for the species *Campylobacter jejuni*, *Campylobacter coli*, *Campylobacter lari* and *Campylobacter upsaliensis* was assessed in the FAM fluorescence channel. Additionally, *Campylobacter jejuni* was assessed in the ROX channel, and *Campylobacter coli* was assessed in the Cy5 channel—in both cases only as either absent or present. Specific amplification of the internal control was analyzed in the HEX channel.

After each run, the results were validated, and a calibration curve was used, resulting in an “SQ” value for each sample analyzed. This was performed in order to convert the SQ value to CFU/g.

### 2.7. Statistical Analyses

Statistical analysis was performed using Minitab^®^ version 21.3—2022 software (Minitab Inc., State College, TX, USA). The results for the level of contamination obtained using the three proposed methodologies, taking into account chilled and frozen turkey cuts, underwent analysis using the Kruskal–Wallis non-parametric test in order to identify significant differences (*p* < 0.05) because the data were categorical and therefore non-parametric. The Anderson–Darling (*p* < 0.005), Ryan–Joiner (*p* < 0.01) and Kolmogorov–Smirnov (*p* < 0.01) tests were also applied in order to evaluate the normality of the data.

The Anderson–Darling (*p* < 0.005), Ryan–Joiner (similar to Shapiro–Wilk, *p* < 0.01) and Kolmogorov–Smirnov (*p* < 0.01) tests were applied in order to evaluate the distribution of the data. All the test results presented a non-normal distribution. Because the data were deemed to be non-normal, the mean and standard deviation were not used in the statistical test; the median was used. The results of the three methodologies were plotted on a chart in order to assess for a possible correction factor, both for the chilled and for the frozen conditions. In order to evaluate the relative truthfulness of the data, the Bland–Altman test with a confidence interval of 95% for the upper and lower control limits was used in order to assess the difference between the reference method ISO 10272-2:2017 [36] and the alternative methods TEMPO^®^ CAM (bioMérieux^®^, Marcy-l’Etoile, France) and rtPCR (Biotecon^®^, Potsdam, Germany).

## 3. Results

Over 12 months, a total number of 416 samples were analyzed, comprising 208 samples of chilled turkey cuts and 208 samples of frozen turkey cuts, including drumstick, breast and wing. During the sample-harvesting period, 104 samples were taken for each season of the year (autumn, winter, spring and summer).

Of the 208 samples of chilled turkey meat that were analyzed, *Campylobacter* spp. was quantified in 11 samples (5.3%) using the ISO 10272-2:2017 [36] reference methodology; 61 (29.3%) samples were quantified using the TEMPO^®^ CAM methodology; and 6 (2.9%) samples were quantified using the rtPCR methodology. Of the 208 samples of frozen turkey meat that were analyzed, *Campylobacter* spp. was quantified in 1 sample (0.5%) using the reference methodology; 44 (21.2%) samples were quantified using the TEMPO^®^ CAM methodology; and 1 sample was quantified (0.5%) using the rtPCR methodology. 

Table 4 displays the mean of quantification results for *Campylobacter* spp. separated by each type of chilled and frozen cut and by the three methodologies that were applied. Table 5 displays the mean and standard deviation of the results in accordance with the temperature condition of the samples. 

### 3.1. Data Evaluation Using the Kruskal–Wallis Test

For the frozen cuts, after the Kruskal–Wallis statistical test was applied to the quantification data, the *p*-value was calculated (*p* = 0.780), which concluded that the quantitative analysis did not show a significant difference (*p* > 0.05) between the reference methodology and rtPCR methods; however, between the reference methodology and TEMPO^®^ CAM methods, the *p*-value was calculated as *p* = 0.000, and the conclusion was that the results presented a significant difference (*p* < 0.05).

### 3.2. Data Evaluation Using the Bland–Altman Test

**Figure 1:**
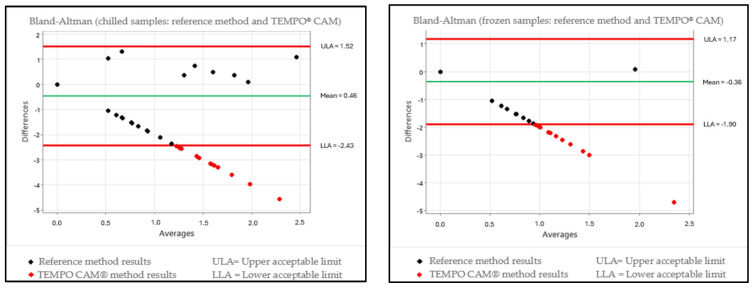
Scatter plot showing the Bland–Altman test with results of the differences between the reference methodology (ISO 10272-2:2017) and the TEMPO^®^ CAM methodology for chilled and frozen samples.

**Figure 2:**
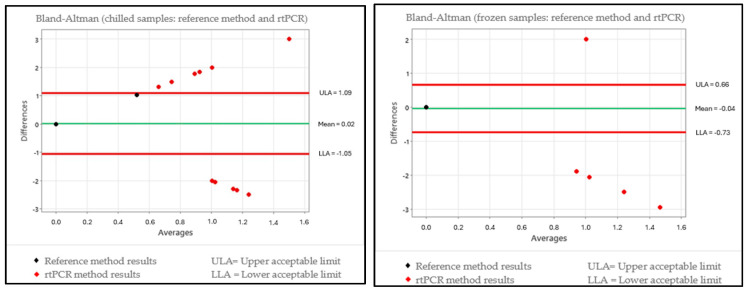
Scatter plot showing the Bland–Altman test with results of the differences between the reference methodology (ISO 10272-2:2017) and the rtPCR Biotecon^®^ methodology for chilled and frozen samples.

In order to assess for relative truthfulness, the data were plotted on a scatter chart for a Bland–Altman test interpretation, which enabled the assessment of the differences in results between the reference methodology ISO 10272-2:2017 [36] and the alternative methodology for each temperature condition. The international methodology ISO 16140-2:2016 [59] was used as the reference, which takes into account an assessment of the results obtained using naturally contaminated samples, where the standard for interpretation that is described in the corresponding standard sets forth that preferably only 1 out of every 20 values for the data obtained should be outside the range of the upper and lower tolerance limits. The charts for each evaluation in accordance with the condition of the samples and the alternative methods are given in Figure 1 and Figure 2.

Each of the groups (chilled and frozen) was plotted on the charts with the 208 samples in order to interpret the Bland–Altman test, and it was established that for satisfactory truthfulness, a maximum of 10 samples could be outside the limits of tolerance interval. The interpretation of Figure 1, which interpolates the alternative method TEMPO^®^ CAM, revealed that for the chilled condition, the method presented 16 samples that were outside the range of the limits of tolerance; meanwhile, for the frozen condition, the method presented 19 samples that were outside the range of the limits of tolerance. In both sample conditions, there were more than 10 deviations each, corroborating the fact that truthfulness was not satisfactory. For evaluating the alternative method of rtPCR Biotecon^®^, Figure 2 was created and showed that, for the chilled samples, there were 13 deviations outside the range of the limits of tolerance, indicating that truthfulness was not satisfactory. However, for the frozen condition, the rtPCR method presented five samples that were outside the range of the limits of tolerance, and it could be characterized as having a good performance.

### 3.3. Comparison of the Kruskal–Wallis and Bland–Altman Test Data

For the comparison of the Kruskal–Wallis and Bland–Altman tests (Table 6), since they are different tests with different criteria, they both possess a difference in the acceptance of the equivalency of methods; however, the results were identical, except for the results of the comparison between the reference method and the rtPCR method for the chilled samples.

### 3.4. Evaluation of Data for Calculation of Correction Factor

**Figure 3:**
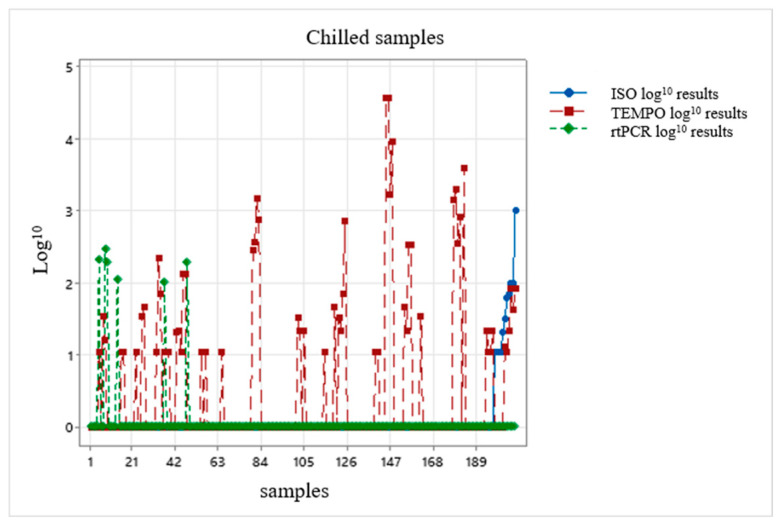
Evaluation of the correction factor for results in log_10_ CFU/g of the reference methodology ISO 10272-2:2017, the alternative methodology TEMPO^®^ CAM and the alternative methodology rtPCR Biotecon^®^ applied to the chilled samples.

**Figure 4:**
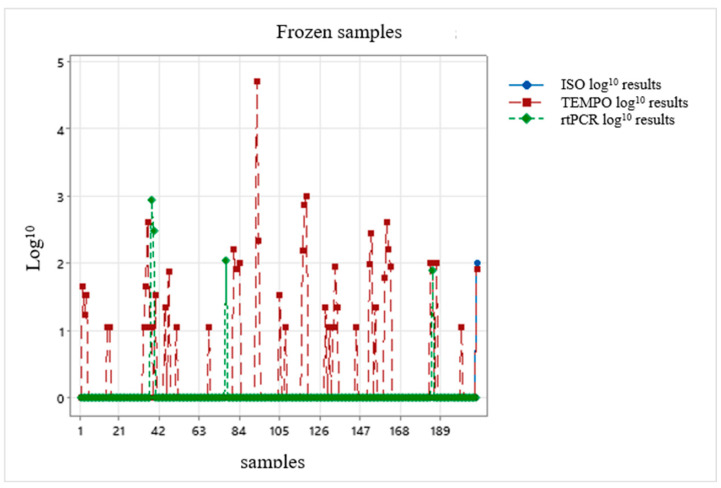
Evaluation of the correction factor for results in log_10_ CFU/g of the reference methodology ISO 10272-2:2017, the alternative methodology TEMPO^®^ CAM and the alternative methodology rtPCR Biotecon^®^ applied to the frozen samples.

The results of the three methodologies were plotted on a chart, taking into account the assessment of a possible correction factor: one chart representing the chilled condition and the other chart representing the frozen condition (Figure 3 and Figure 4). 

After the results were interpolated, they were unable to provide any correction factor for the alternative methodologies because the results presented a non-normal distribution (Figure 3 and Figure 4); in other words, the data were highly dispersed and did not allow a suitable value to exist.

## 4. Discussion

The three methodologies tested in the present study—namely the ISO 12720:2017 [36], which was the reference methodology, and the alternative methodologies TEMPO^®^ CAM (bioMérieux^®^) and rtPCR (Biotecon^®^)—differed in the cost–benefit requirements, ease of execution and time of execution. 

The differences became more relevant when comparing the results of the quantifications of the present study because the quantitative analysis for both of the storage temperature conditions for turkey meat (chilled and frozen) in the Kruskal–Wallis test did not indicate a significant difference (*p* > 0.05) between the ISO 10272-2:2017 [36] and the rtPCR methods; however, between the ISO 10272-2:2017 [36] and the TEMPO^®^ CAM methods, the results did present a significant difference (*p* < 0.05). For the Bland–Altman test, the TEMPO^®^ CAM methodology presented results outside the expected range for both chilled and frozen turkey cuts, whereas the rtPCR methodology presented a result outside the expected range for the chilled turkey cuts and a result within the expected range for the frozen turkey cuts.

The results of the alternative methodology TEMPO^®^ CAM applied to chilled turkey cuts displayed a mean and standard deviation of 1.09 ± 1.15 log_10_ CFU/g, while for the reference methodology, the mean and standard deviation were 0.17 ± 0.53 log_10_ CFU/g. For the frozen cuts, the alternative methodology TEMPO^®^ CAM displayed a mean and standard deviation of 0.75 ± 0.99 log_10_ CFU/g, while for the reference methodology, the mean and standard deviation were 0.02 ± 0.20 log_10_ CFU/g. The difference between the two methodologies without taking into account the standard deviation was 0.92 log_10_ CFU/g for the chilled turkey cuts and 0.73 log_10_ CFU/g for the frozen cuts. The means and the standard deviations among the methodologies were higher, corroborating the statistical result of the Kruskal–Wallis test, which showed there was a significant difference (*p* < 0.05), and the Bland–Altman test, which also presented results outside the expected range of values. The difference in the means of quantifications may indicate an unequal distribution of organisms that is frequently observed in naturally contaminated samples because they are not homogenized, as opposed to artificially contaminated samples [60]. However, this study was performed based on the same sample; in other words, the aliquots for inoculating the TEMPO^®^ CAM card and the mCCDA plate of the reference methodology were identical, nullifying the corresponding variable.

Because this is an alternative methodology that has only recently become available for quantifying *Campylobacter* spp., a sufficient number of scientific studies presenting data to enable conclusions in the present study on TEMPO^®^ CAM were not found, and validation information was limited to NF Validation—Certificate Number: BIO 12-/43-04/20 [61]. Therefore, scientific studies carried out using other species of micro-organisms available within the TEMPO^®^ methodology were evaluated. Crowley et al., in a collaborative inter-laboratory study for AOAC, comparing the tests of mesophilic aerobic micro-organisms using the TEMPO^®^ TVC method and the Petrifilm^®^ TVC method in a ground beef matrix, obtained a significant statistical difference (*p* < 0.05) in samples with a low level of contamination [60]. In a later study by Crowley et al., in which the TEMPO^®^ EC method was compared against the AOAC 966.24 method, both of which were testing for *Escherichia coli* in a ground beef matrix, a statistically significant difference (*p* < 0.05) was found between the methods, specifically at low (50 CFU/g) and medium (500 CFU/g) levels of contamination [62]. In the present study, the results we obtained did not indicate a possible trend of low, medium or high levels because the results presented variations at all concentrations of the micro-organisms quantified.

In a pasteurized milk matrix, the assays for indicator micro-organisms using the Petrifilm^®^ and TEMPO^®^ TVC, TEMPO^®^ CT and TEMPO^®^ EC methods were compared, in which the mean counts for mesophilic aerobic micro-organisms, coliforms at 45 °C and *Escherichia coli* presented a statistically significant difference (*p* < 0.05), showing the lower level of performance of the TEMPO^®^ methodology for these assays [63]. The results presented in these three studies corroborated the statistical difference (*p* < 0.05) that was found for the TEMPO^®^ CAM method in the present study.

The TEMPO^®^ method is an automated MPN methodology, whereas the reference method is a direct plate-count assay [64]. The principle underlying an MPN method is inherently more variable than a direct plate-count methodology because the MPN method produces a mathematical estimation of the micro-organisms that are present rather than a direct measurement of the organism counts [64]. It is a reasonably old method (introduced in c. 1915) that is still widely used, but its results are rather imprecise, since it allows only an estimate of the number of micro-organisms [65]. On the other hand, liquid media stimulate the recovery of stressed bacterial cells after exposure to several physical treatments, such as conservation at cold temperatures [66], suggesting that this is one of the possible reasons why the TEMPO^®^ CAM method quantified more samples over the course of the entire study.

Taking into account the alternative methodology of rtPCR (Biotecon^®^), the results for chilled turkey cuts displayed a mean and standard deviation of 0.13 ± 0.53 log_10_ CFU/g, while for the reference methodology, the mean and standard deviation were 0.17 ± 0.53 log_10_ CFU/g. For the frozen cuts, the alternative methodology of rtPCR (Biotecon^®^) displayed a mean and standard deviation of 0.09 ± 0.46 log_10_ CFU/g, while for the reference methodology, the mean and standard deviation were 0.02 ± 0.20 log_10_ CFU/g. The difference between the two methodologies without taking into account the standard deviation was 0.04 log_10_ CFU/g for the chilled turkey cuts and 0.07 log_10_ CFU/g for the frozen cuts. The mean and the standard deviation between the methodologies were very close, corroborating the statistical result of the Kruskal–Wallis test, which showed there was no significant difference (*p* > 0.05), and the Bland–Altman test, which gave a result within the expected values only for the frozen samples. Perdoncini et al. performed a study in which several steps of the slaughter process of broiler chickens were evaluated and, by comparing the reference methodology against rtPCR, also failed to obtain a significant difference (*p* > 0.05) between the two methods [66]. Likewise, Lazou et al., comparing the rtPCR and reference methodologies in a lamb meat matrix, found no significant difference (*p* > 0.05), and therefore, it may be an alternative method for use [67]. The rtPCR method correlated significantly with the plate-count methodology.

Of the 11 samples quantified in the rtPCR methodology, 10 presented results in the exponential range of micro-organism concentration at 10^2^, and only 1 was found in the 10^1^ exponential range of concentration. In the reference methodology, out of the 12 results that were obtained, only 4 were exponential quantifications between 10^2^ and 10^3^, and the other 8 samples presented an exponential result of 10^1^. According to the specifications of the manufacturer of the rtPCR test (Biotecon^®^), the analytical kit was validated for the exponential range of 10^3^ in accordance with the European legislation. According to the manufacturer’s guidance, the kit was validated up to the exponential micro-organism concentration range of 10^1^ by internal validation using certified reference material (MRC Microbiologics^®^) for the species *Campylobacter jejuni* subsp. jejuni, ATCC^®^ reference number 33560TM.

Comparing the rtPCR results with the detection ranges, the study suggests that the methodology demonstrates a limited quantification condition for the exponential range of 10^−2^. Papic et al. suggest in their study that PCR-based methods require a relatively high level of contamination for reliable detection and quantification [31], as do Castañeda-Gulla et al., where they describe that the limit of detection of the rtPCR method shows limitations in samples containing low micro-organism counts [68]. According to Irwin et al., a low concentration of target micro-organisms may impede reliable detection and quantification, and the quantification of samples with a low concentration (<1000 CFU) in complex samples is made difficult because of inherent differences in the efficiency of DNA amplification [69].

In 10 samples (6 of which were chilled and 4 frozen), the rtPCR method quantified *Campylobacter* spp., while the same 10 samples tested with the reference methodology did not quantify the micro-organism. During assays using the reference methodology, it was found that there was growth of the characteristic colonies, but they were not confirmed by the tests recommended in the methodology for *Campylobacter* spp. According to Repérant et al., the growth of competitive microbiota on mCCDA plates after enrichment may lead to a false negative confirmation for a positive sample [70]. Not all cells can be recovered via conventional cultivation techniques because of special growth requirements and the status of cells that are viable but non-cultivable [36]—a survival strategy used by many micro-organisms [71]. In this case, the PCR technique plays an important role in identifying typical bacterial cultures that exist in viable, albeit non-cultivable, coccoid forms (for example, *Campylobacter* spp.) and which are often lost when using the conventional method [72], thus suggesting that the 10 results obtained using the rtPCR may have been due to the fact that these cells existed in coccoid forms, whereby it was only possible to detect the micro-organism through DNA extraction, since it was not cultivable in this format, and therefore, it could not be quantified using the reference methodology.

The molecular methods for detecting and quantifying pathogens are rapid, selective and accurate; however, the inability to differentiate viable cells from dead cells hinders their use in the food industry [31,69]. To mitigate the influence of dead cells, the rtPCR method used in the present study deployed reagent D (Biotecon^®^) in the extraction step, the specific function of which is to remove dead cells, so that only viable cells can be quantified. It is thus possible to state that results that did not present quantification via rtPCR for concentrations above 100 CFU/g were truthful with the reference methodology results.

The Kruskal–Wallis and Bland–Altman tests revealed that the rtPCR method presented satisfactory results for the frozen samples in both, and for the chilled samples, the Kruskal–Wallis test was also satisfactory; these results underscore the fact that the rtPCR method is reliable and could be used. In light of the results presented via the quantification of *Campylobacter* spp., it was suggested as a safety measure that the micro-organism should be confirmed using the reference methodology, thereby enabling an enhancement of the procedure. 

Owing to the results obtained through the use of the three methodologies that were applied, the sample matrix was suggested to be a possible variable in the analytical process. The sample type and the analytical method used influence the quantitative results on the *Campylobacter* spp. colonization level [73]. The matrix effects may produce unexpected results [74], and these effects may not be recognized by an internal validation procedure, and they may lead to false negative results [75]. The turkey carcass chilling process prior to cutting involves a pre-chilling step in a chiller, and then, the carcasses remain in a chiller chamber under an air flow until attaining the minimum temperature of 4 °C at which they may be processed for producing cuts and subsequent freezing. The effect of the chilling process on the micro-organisms is to curb or inhibit their growth [14], and one of the main reasons for this is the fact that some of the micro-organisms lose their culturing viability owing to cold stress [26], as with freezing, which induces injury and oxidative stress to the micro-organism [76].

Brazil is among the countries that have succeeded in drastically reducing food insecurity, especially due to the substantial increase in the country’s agricultural productivity [77]. Brazilian poultry farming has shown high growth rates as a result of modernization, adequate poultry management and integrated production, which have contributed to technical excellence at all stages of the production chain [78]. Carcass washing combined with control programs, such as Good Manufacturing Practices (GMP), Hygienization Procedures (HP) and Hazard Analysis and Critical Control Points (HACCP), enable an efficient and safe microbiological control [22].

Josefsen et al., studying *Campylobacter* spp. in chicken carcasses using rtPCR, showed that the use of a rinsing procedure is efficient in recovering 27–47% of the micro-organism; however, when double rinsing is used, this efficiency rises to 55–94% recovery of cells [79]. In the present study, destructive sampling was used, removing the sample portion from different parts of each cut of turkey meat until attaining a representative portion (25 g), and this factor suggests that it may have influenced the detection of the micro-organism [44]. For the molecular methods, the low concentration of target micro-organisms may impede reliable detection and quantification in naturally contaminated samples [65]. The intrinsic sensitivity of molecular technologies is a positive feature, but owing to the sample preparation steps and to the low entry volume used in the methodology, it has been shown that the sensitivity of the methodology for diagnostic purposes in foods ranges from 100 to 1000 CFU/mL [79]; in other words, a more concentrated entry material could be obtained for DNA extraction in addition to smaller volumes for DNA, or DNA polymerase, dilution for detection in the proposed levels [31].

International validations of analytical methods must be evaluated and interpreted cautiously, since they do not take into account the regional variations in the microbial ecology of foods: these regional variations, both of the microbiota and of the food matrix, or even certain intrinsic characteristics of the foods, may influence the performance of the alternative methods [80]. The performance of a methodology in diagnosing foods is widely affected by factors such as the type of food and the associated microbial ecology and/or the stressing conditions that the foodstuff imposes on the target micro-organism [79]. It was not possible—probably owing to the variations and factors reported by Tavolaro et al. and Jasson et al., 2010 [79,80], when the results of the three methodologies were plotted for both the chilled and frozen conditions—to suggest a correction factor for the alternative methodologies because the results presented a non-normal distribution. The search for a correction factor is of the utmost importance to make rapid methodologies feasible for use in the meat industry.

## 5. Conclusions

In the comparison of the methods, it is possible to state that the ISO 10272-2:2017 [36] reference methodology is one of the most suitable standards for bacteriological culturing for the *Campylobacter* spp. assay, and it is within the necessary limits of quantification for controlling the performance of slaughter processes both for monitoring and for taking actions to address cases of possible deviations in health and food safety criteria. Compared with the reference methodology, the TEMPO^®^ methodology using the TEMPO^®^ CAM kit did not present satisfactory results in any of the evaluations and is not recommended for use in the turkey meat matrix. In the comparison with rtPCR Biotecon^®^, the performance was deemed satisfactory because there was a good correlation in the data, above all in those that were not quantified, and its use is recommended for the turkey meat matrix, although the fact that it presented a limited condition for quantification in the exponential range of 10^2^ was a limiting factor that the user should assess prior to implementation. As a measure of safety and reliability, it is recommended that samples with quantification of *Campylobacter* spp. in rtPCR are confirmed subsequently with the reference methodology.

The validation of alternative methodologies to be implemented is of the utmost importance and must present results that meet the requirements of the analytical applications and that ensure the credibility of the data during routine use to replace the reference methodology. Given the need for meat-producing companies to make use of more rapid results, it is essential that the alternative methodologies are implemented and adapted to a wide range of sample matrices produced by each operator because they add great value to the microbiological routine by bringing rapidity and automation to the process, providing solutions for current and future demands within increasingly technical businesses in which the capacity increases constantly.

The present study is limited to the turkey meat matrix, and it is therefore recommended that further studies are performed using the same matrix with a greater sampling volume by means of rinsing procedures and above all using artificially fortified samples in parallel to define a possible suitable correction factor. It is also essential that research into other matrices is extended in order to verify whether the behavior of the alternative methodologies is similar to the present study or whether there is an enhancement in performance detectable in the results.

## Figures and Tables

**Table 1:** Publications reporting the indices of *Campylobacter* spp. found in turkey cuts and carcasses. These indices were used to define the number of samples to be taken during the study, regardless of the type of methodology used in each publication.

Reference	Country	Average Value of Prevalence in Turkeys (%)
[48]	Poland	38.0
[10]	Europe	65.3
[49]	Canada	38.2
[50]	Turkey	45.6
[51]	Iran	28.8
[52]	Iran	32.8
[53]	Germany	29.2
[54]	Ireland	37.5

**Table 2:** Preparation of the Foodproof (Biotecon^®^, Potsdam, Germany) mix for subsequent preparation of the PCR test tubes in accordance with the order shown in the table.

Component	Volume ^a^
Foodproof *Campylobacter* Master Mix	18 μL
Foodproof *Campylobacter* Enzyme Solution	1 μL
Foodproof *Campylobacter* Internal Control	1 μL

^a^ Total volume of 20 μL per sample.

**Table 3:** Interpretation chart of the rtPCR results after finalization of the run to quantify *Campylobacter* spp. and to identify the most significant species.

FAM	ROX	Cy5	HEX	Result
+	-	-	+ or -	*Campylobacter* spp. detected
+	+	-	+ or -	Positive for *Campylobacter* spp. and *C. jejuni*
+	-	+	+ or -	Positive for *Campylobacter* spp. and *C. coli*
+	+	+	+ or -	Positive for *Campylobacter* spp., *C. jejuni* and *C. coli*
-	-	-	+	Negative for *Campylobacter* spp., *C. jejuni* and *C. coli*
-	-	-	-	Result invalid

**Table 4:** Mean of the quantitative results for *Campylobacter* spp. in chilled and frozen turkey cuts obtained using the reference methodology ISO 10272-2:2017 and the alternative methodologies TEMPO^®^ CAM and rtPCR Biotecon^®^ throughout the period from March 2022 to April 2023.

*Campylobacter* spp. Count log_10_ CFU/g (Mean)			
Material	ISO 10272-2 Method	TEMPO^®^ CAM Method	rtPCR Method (Biotecon^®^)
Chilled turkey wings	0.09	0.51	0.14
Frozen turkey wings	0.00	0.38	0.04
Chilled turkey drumsticks	0.10	0.54	0.04
Frozen turkey drumsticks	0.04	0.39	0.09
Chilled turkey breast	0.07	0.54	0.04
Frozen turkey breast	0.01	0.37	0.07

Note: All results (100%) were taken into account to calculate the means (416 samples).

**Table 5:** Mean and standard deviation of the quantitative results for *Campylobacter* spp. in chilled and frozen turkey cuts obtained using the reference methodology ISO 10272-2:2017 and the alternative methodologies TEMPO^®^ CAM and rtPCR Biotecon^®^.

	Quantification Results (Mean ± SD)		
	ISO 10272-2	TEMPO^®^ CAM	rtPCR Biotecon^®^
	log_10_ CFU/g	log_10_ CFU/g	log_10_ CFU/g
Chilled cuts	0.17 ± 0.53	1.09 ± 1.15	0.13 ± 0.53
Frozen cuts	0.02 ± 0.20	0.75 ± 0.99	0.09 ± 0.46
Chilled and frozen cuts	0.09 ± 0.41	0.91 ± 1.18	0.11 ± 0.49

SD = Standard Deviation. The values of each sample were taken into account to perform the calculations, regardless of the temperature condition in which quantification was obtained for at least one of the applied methodologies. Samples were not taken into account where quantification did not occur in the three methodologies that were applied.

**Table 6:** Comparison of the results for *Campylobacter* spp. using the reference methodology ISO 10272-2:2017, the alternative methodology TEMPO^®^ CAM and the alternative methodology rtPCR Biotecon^®^ obtained using turkey cuts (interpreted using the Kruskal–Wallis and Bland–Altman tests).

Condition of Sample	Comparison of Methods	Kruskal–Wallis Test	Bland–Altman Test
Chilled	ISO vs. Tempo	Statistically different	Outside expected range
	ISO vs. rtPCR	Statistically equal	Outside expected range
Frozen	ISO vs. Tempo	Statistically different	Outside expected range
	ISO vs. rtPCR	Statistically equal	Within expected range

## Data Availability

The original contributions presented in the study are included in the article, further inquiries can be directed to the corresponding author.
